# Key to better outcomes in stroke intervention: early versus complete reperfusion in first pass recanalization

**DOI:** 10.1007/s00415-025-13235-5

**Published:** 2025-07-11

**Authors:** Alexander Heitkamp, Sophie-Maria Hierholzer, Christian Heitkamp, Laurens Winkelmeier, Lukas Meyer, Matthias Bechstein, Vincent Geest, Gabriel Broocks, Caspar Brekenfeld, Fabian Flottmann, Maximilian Schell, Götz Thomalla, Tobias Faizy, Jens Fiehler, Helge C. Kniep, Susanne Gellißen, for the German Stroke Registry -Endovascular Treatment (GSR-ET)

**Affiliations:** 1 Department of Diagnostic and Interventional Neuroradiology, University Medical Center Hamburg-Eppendorf, Martinistraße 52, 20246 Hamburg, Germany; 2 Department of Neuroradiology, HELIOS Medical Center, Campus of MSH Medical School Hamburg, Schwerin, Germany; 3 Department of Neurology, University Medical Center Hamburg-Eppendorf, Hamburg, Germany; 4 Neuroendovascular Program, Department of Radiology, University Hospital Muenster, Muenster, Germany

**Keywords:** mTICI, Ischemic stroke, First pass, Mediation analysis, Mechanical thrombectomy

## Abstract

**Background and purpose:**

First pass (FP) recanalization, defined as achieving mTICI 2b or higher in a single thrombectomy attempt, has been linked to better functional recovery in acute ischemic stroke patients. This study aimed to investigate whether the benefits of FP are primarily driven by higher rates of complete reperfusion (mTICI 3) or by faster procedure times.

**Methods:**

Data from 3707 patients with middle cerebral artery occlusion and successful recanalization (mTICI 2b or higher) were extracted from the prospectively designed German Stroke Registry (2015–2021). Good functional outcomes were defined as a modified Rankin Scale (mRS) score of ≤ 2 at 90 days. Mediation analysis was used to evaluate the extent to which complete reperfusion (mTICI 3) and shorter groin puncture to recanalization time contributed to improved outcomes.

**Results:**

FP recanalization was associated with significantly better functional outcomes: 46.9% of FP patients achieved an mRS ≤ 2 compared to 37.2% in the multi-pass group. Mediation analysis showed that only 14% of the improved outcomes with FP were explained by higher mTICI 3 rates, while 37% were attributed to faster recanalization times.

**Conclusion:**

The improved outcomes associated with FP recanalization are primarily driven by the speed of reperfusion rather than the degree of complete recanalization. This highlights the importance of minimizing procedure times and the number of thrombectomy attempts. Strategies aimed at optimizing treatment workflows and improving device design to prioritize early and efficient reperfusion after the FP are critical to improving patient outcomes (ClinicalTrials.gov identifier: NCT03356392).

## Introduction

Endovascular thrombectomy (EVT) is widely recognized as the standard treatment for acute ischemic stroke caused by large-vessel occlusion in the anterior circulation [1]. First pass (FP) recanalization with a modified Thrombolysis in Cerebral Infarction (mTICI) score of 2b-3 has been shown to improve functional outcome in patients with ischemic stroke undergoing EVT, compared to multi-pass procedures [2–5]. Therefore, the FP rate is becoming increasingly important in the design and evaluation of thrombectomy devices [6].

Pathophysiological factors contributing to improved functional outcome after FP recanalization may include reduced mechanical stress on vessel walls and shorter time to recanalization. Additionally, higher rates of complete recanalization mTICI 3 were observed in FP recanalization compared to multi-pass procedures [7], and beneficial effects of mTICI 3 compared to mTICI 2b have been reported in previous studies [8, 9]. Notably, the combination FP recanalization and complete recanalization (mTICI 3) has been shown to significantly improve functional outcome, a phenomenon often referred to as the true first-pass effect (T-FPE) [2, 10, 11].

Despite these findings, it remains unclear whether the improved outcomes associated with FP recanalization are primarily driven by faster recanalization, reduced mechanical stress on vessel walls, less thrombus fragmentation or by the achievement of higher rates of complete recanalization (mTICI 3). This raises the question of whether procedure and device optimization should prioritize rapid FP recanalization at any level mTICI ≥ 2b, or if complete recanalization mTICI 3 is the main determinant of improved outcome after FP recanalization.

This study aims to assess whether higher rates of complete reperfusion mTICI 3 or faster procedure times are the primary factor contributing to improved outcomes after FP recanalization. We hypothesize that faster recanalization is the main determinant of the improved outcomes observed after FP recanalization.

## Materials and methods

### Study design

We performed a retrospective multicenter cohort study with patients enrolled in the German Stroke Registry—Endovascular Treatment (GSR) between May 1, 2015, and December 31, 2021. The GSR is an ongoing, open-label, industry-independent, prospective registry that includes patients with acute ischemic stroke due to large vessel occlusion who received endovascular thrombectomy at one of 25 comprehensive stroke centers in Germany (ClinicalTrials.gov identifier: NCT03356392). A detailed description of the GSR-ET study design has been published [12, 13]. Ethical approval was obtained from the ethics committee of the Ludwig Maximilian University in Munich, Germany (689-15), and local ethics committees of each participating center approved the contribution of fully anonymized data to the registry. The study adheres to the Strengthening the Reporting of Observational Studies in Epidemiology (STROBE) guidelines [14].

### Study cohort

**Fig. 1:**
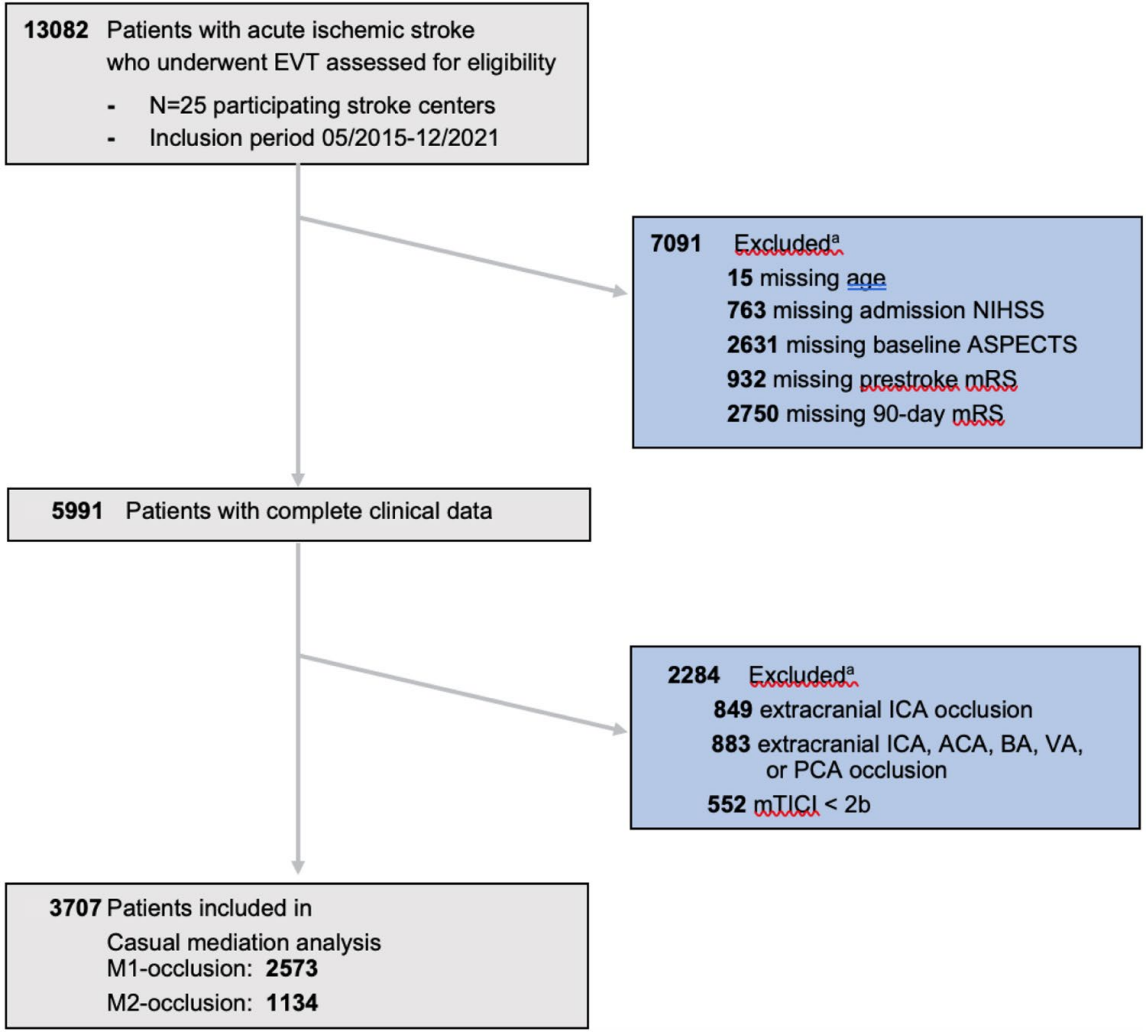
Flow chart of patient inclusion and exclusion criteria. Abbreviations: EVT, Endovascular Thrombectomy; NIHSS, National Institutes Health Stroke Scale; ASPECTS, Alberta Stroke Program Early CT Score; mRS, modified Rankin Scale; ACA, Anterior Cerebral Artery; ICA, Internal Carotid Artery; MCA, Middle Cerebral Artery; BA, Basilar Artery; VA, Vertebral Artery; PCA, Posterior Cerebral Artery; M1-occlusion, first segment middle cerebral artery occlusion; M2-occlusion, second segment middle cerebral artery occlusion; mTICI, modified thrombolysis in cerebral infarction. ^a^Multipe selection of patients possible

All patients prospectively enrolled in the GSR from May 2015 to December 2021 were screened. Inclusion criteria were middle cerebral artery occlusions, successful recanalization with mTICI ≥ 2b, and the availability of relevant clinical data (availability sampling). An overview of the inclusion and exclusion criteria is given in Fig. 1.

### Clinical and radiological assessment

Clinical and imaging characteristics were used as reported by the local investigators at each participating center, including baseline clinical characteristics, radiological information, procedural and outcome parameters.

### Study outcome measures

Primary endpoint was good functional outcome, defined as a modified Rankin Scale (mRS) score of ≤ 2 at 90 days. Additional endpoints were successful recanalization defined as mTICI 2b-3, complete recanalization defined as mTICI 3, FP recanalization, defined as successful recanalization mTICI 2b-3 after one single pass and the time from groin puncture to recanalization measured in minutes.

### Statistical analysis

Descriptive statistics were used to compare characteristics between patients with FP versus multi-pass recanalization. Categorical variables were reported as counts and percentages and continuous variables were reported as median and interquartile range (IQR) unless otherwise stated. Statistical tests between subgroups were conducted using Pearson’s chi-squared test for categorical variables and Wilcoxon rank-sum test for continuous variables.

Multivariable logistic and linear regression analysis was performed to identify independent predictors of good functional outcome, complete recanalization and time from groin puncture to recanalization. Relevant factors were determined using backward variable selection procedures based on the Akaike information criterion (AIC). For the mediation models, all factors significantly associated with good functional outcome and all factors significantly associated with full recanalization and groin puncture to recanalization time in logistic and linear regression models were selected. The following variables were included in the models: Age, sex, baseline mRS, baseline National Institutes of Health Stroke Scale (NIHSS), comorbidity diabetes, comorbidity dyslipidemia, Alberta Stroke Program Early CT Score (ASPECTS), FP, full recanalization, groin puncture to recanalization time and adverse treatment events of clot migration embolization and intracranial hemorrhage (intraprocedural contrast agent extravasation). Adjusted Odds ratios (aOR) with 95% confidence intervals (CI) and *p*-values were calculated.

Mediation analysis was conducted twice: (Model A) To determine the extent to which FP-related improvements in functional outcomes after 90 days can be attributed to achieving mTICI 3 compared to mTICI 2b recanalization. (Model B) To determine the extent to which FP-related improvements in functional outcomes after 90 days can be attributed to a shorter groin puncture to recanalization time. Regression models used in this analysis followed the approach described by Mackinnon and Dwyer [15]. The mediation analysis adhered to the guidelines established by Baron and Kenny [16] and utilized algorithms from Imai and Keele [17, 18]. The effects were quantified using the average causal mediation effect (ACME) and the average direct effect (ADE), with FP recanalization (vs. multi-pass recanalization) as the treatment/exposure variable, and complete recanalization (mTICI 3) (Model A) and groin puncture to recanalization time (Model B) as the mediator. Good functional status at 90 days was defined as the outcome variable. Treatment effects were expressed as the absolute increase in the probability of a good functional outcome, measured in percentage points (pp), equivalent to absolute risk reduction (ARR). Although the interventionalist cannot actively choose between FP and multi-pass recanalization during the procedure—making the term “treatment” somewhat imprecise—we have retained this terminology for consistency with standard mediation analysis, while acknowledging that it may alternatively be described as an “exposure effect.” Confidence intervals of the mediation analysis were obtained using a Quasi-Bayesian Monte Carlo method-based approximation [19]. A sensitivity analysis for the mediation effect with complete recanalization (Model A) and groin puncture to recanalization time (Model B) as mediator was conducted based on subcohorts differentiating patients with M1 and M2 occlusions. All analyses were carried out using R 4.4.1 with the mediation package 4.5.0 [19]. All presented results of the mediation analysis were statistically significant with a *p-*value < 0.05 unless otherwise stated.

### Data availability

Data are not publicly available. Data that support the findings of this study are available upon reasonable request after approval of the ethics committee and all participating centers.

## Results

### Study cohort baseline characteristics

The analysis included 3707 patients prospectively enrolled between May 2015 and December 2021 with middle cerebral artery stroke and successful recanalization mTICI 2b-3. Among these, FP recanalization was observed in 1914 patients (51.6%), while 1793 patients (48.4%) underwent multi-pass recanalization. The median age was 77 years in the FP cohort and 76 years in the multi-pass cohort. Overall, 52% of the patients were female and 48% were male. FP recanalization was associated with significantly better functional outcomes: 46.9% of FP patients achieved an mRS ≤ 2 compared to 37.2% in the multi-pass group (*p* < 0.001). Complete recanalization mTICI 3 was achieved in 2301 patients (62% of the full cohort) with 920 cases in the multi-pass group (51% of the multi-pass subcohort) compared to 1381 cases in the FP group (72% of the FP subcohort) (*p* < 0.001). The groin puncture to recanalization time was significantly lower in the FP cohort compared to the multi-pass cohort (29 min vs. 51 min; *p* < 0.001). A total of 1566 patients achieved a good functional outcome, with 898 (57%) in the FP group and 668 (43%) in the multi-pass group. In patients with complete recanalization mTICI 3, 663 out of 1381 patients (48%) with FP recanalization achieved a good functional outcome, compared to 384 out of 933 patients (41.2%) in the multi-pass cohort. All reported adverse treatment events, including dissections (3% vs. 1%, *p* < 0.05), vasospasms (6% vs. 4%, *p* < 0.05), clot migration embolization **(**5% vs. 1%, *p* < 0.001) and intracranial hemorrhages (4% vs. 2%, *p* < 0.001), occurred at higher rates in the multi-pass group compared to the FP group (Tables 1**, **2).

**Table 1:** Study cohort clinical characteristics

	Successful recanalization mTICI 2b-3			
	Multi-pass (N = 1793)	First pass (N = 1914)	Total (N = 3707)	*p*-value
Age (years), median (IQR)	76; (65–83)	77; (68–83)	77; (66–83)	0.002
Sex				0.127
Female	936 (52%)	1047 (55%)	1983 (53%)	
Male	857 (48%)	867 (45%)	1724 (47%)	
Comorbidities				
Antithrombotic medication	782 (44%)	818 (43%)	1600 (43%)	0.59
Arterial hypertonus	1354 (76%)	1470 (77%)	2824 (76%)	0.358
Diabetes mellitus	382 (21%)	446 (23%)	828 (22%)	0.006
Dyslipidemia	731 (41%)	873 (46%)	1604 (43%)	0.003
Atrial fibrillation	750 (42%)	886 (46%)	1636 (44%)	0.145
Baseline characteristics				
pmRS, median (Min–Max)	0 (0–5)	0 (0–5)	0 (0–5)	0.258
NIHSS, median (IQR)	14 (8–18)	14 (9–18)	14 (9–18)	0.874
Imaging characteristics				
Imaging infarct side				0.751
Bilateral	6 (0%)	6 (0%)	12 (0%)	
Left	931 (52%)	987 (52%)	1918 (52%)	
Left, not applicable	0 (0%)	1 (0%)	1 (0%)	
Right, not applicable	0 (0%)	1 (0%)	1 (0%)	
Right	855 (48%)	917 (48%)	1772 (48%)	
Imaging aspects, median (IQR)	9 (7–10)	9.0 (8–10)	9.0 (8–10)	0.011
M1/M2 occlusion				0.212
M1	1227 (68%)	1346 (70%)	2573 (69%)	
M2	566 (32%)	568 (30%)	1134 (31%)	
Treatment characteristics				
Intravenous thrombolysis	879 (49%)	983 (51%)	1862 (50%)	0.155
Anesthesia				< 0.001
Beginning with local change to general anesthesia	65 (4%)	40 (2%)	105 (3%)	
Conscious sedation with local anesthesia	419 (24%)	527 (28%)	946 (26%)	
General anesthesia	1273 (72%)	1300 (70%)	2573 (71%)	
Number of passages, mean (IQR)	2.9 (2–3)	1.0 (1–1)	1.9 (1–2)	< 0.001
Time groin puncture to recanalization, min	51 (34–75)	29 (20–40)	37 (25–59	< 0.001
Full recanalization (mTICI 3)	920 (51%)	1381 (72%)	2301 (62%)	< 0.001
Adverse treatment events				
Device malfunction	10 (1%)	6 (0%)	16 (0%)	0.257
Dissection/Perforation	45 (3%)	26 (1%)	71 (2%)	0.011
Clot migration/embolisation	88 (5%)	13 (1%)	101 (3%)	< 0.001
Intracranial hemorrhage	66 (4%)	30 (2%)	96 (3%)	< 0.001
Vasospasm	107 (6%)	74 (4%)	181 (5%)	0.003
Outcome measures				
90-day mRS score, mean (IQR)	3.4 (1–6)	2.9 (1–5)	3.1 (1–5)	< 0.001
mRS difference, mean (IQR)	2.6 (1–4)	2.2 (1–3)	2.4 (1–4)	< 0.001
Good outcome (mRS <= 2)	668 (37%)	898 (47%)	1566 (42%)	< 0.001

Characteristics were compared between subgroups with the use of either Wilcoxon rank-sum test for continuous variables or Pearson’s chi-square test for categorical variables

Results are reported as n (%) unless otherwise stated

*mRS* modified Rankin Scale, *NIHSS* National Institutes of Health Stroke Scale, *IQR* interquantile range, *M1 occlusion* first segment middle cerebral artery occlusion, *M2 occlusion* second segment middle cerebral artery occlusion

**Table 2:** Outcome characteristics for clinical study cohort

		Good functional outcome mRS 0–2 at day 90						
		Yes			No			
		mTICI2b	mTICI3	Sum	mTICI2b	mTICI3	Sum	Sum
FP	Yes	235 (6%)	663 (18%)	898 (24%)	298 (8%)	718 (19%)	1016 (27%)	1914 (52%)
	No	284 (8%)	384 (10%)	668 (18%)	589 (16%)	536 (14%)	1125 (30%)	1793 (48%)
		519 (14%)	1047 (28%)	1566 (42%)	887 (24%)	1254 (34%)	2141 (58%)	3707 (100%)

Results are reported as n (%)

*FP* first pass, *mTICI* modified thrombolysis in cerebral infarction

### Logistic regression models

**Fig. 2:**
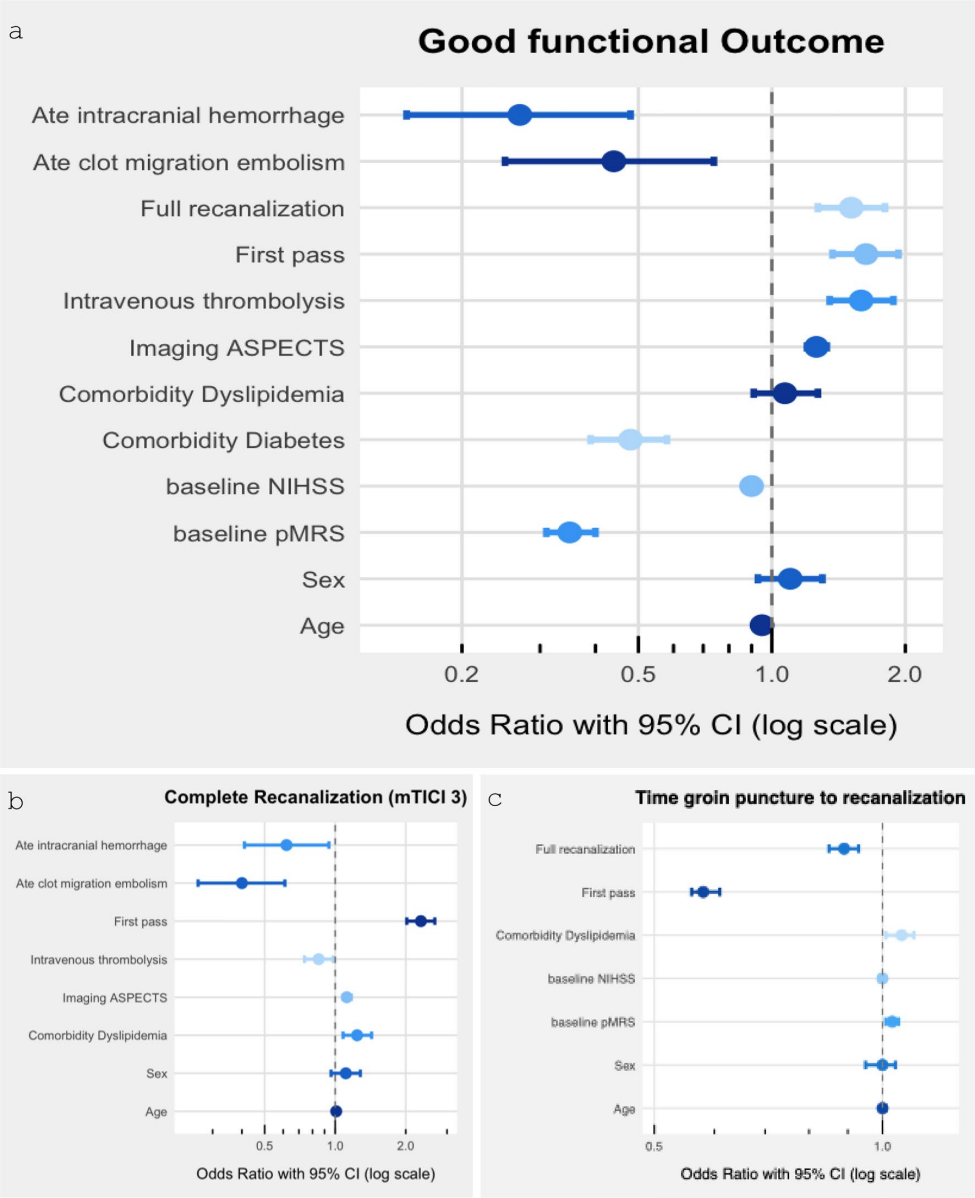
Logistic (**a, b**) and linear (**c**) multivariable regression model results. Multivariable regression models of (adjusted) odds ratios and 95% confidence interval in multivariable and linear logistic regression models of relevant predictors for **a** good functional outcome and **b** complete recanalization and **c** time groin puncture to recanalization (min). Abbreviations: NIHSS, National Institutes Health Stroke Scale; ASPECTS, Alberta Stroke Program Early CT Score; pmRS, premorbid modified Rankin Scale; CI, confidence interval; Ate, adverse treatment event

Results of multivariable logistic regression analysis suggest that probability of good outcome was significantly associated with FP recanalization (aOR = 1.19, *p* < 0.001), full recanalization mTICI 3 (aOR = 1.32, *p* < 0.001) and procedure time (aOR = 0.67, *p* < 0.001). Regression models were controlled for pre-stroke mRS (aOR = 0.31, *p* < 0.001), diabetes mellitus (aOR = 0.52, *p* < 0.001), ASPECTS (aOR = 1.21, *p* < 0.001), intravenous thrombolysis (aOR = 1.59, *p* < 0.001), and intracranial hemorrhage (aOR = 0.35, *p* < 0.001). In the mediator pathway of Model A, full recanalization mTICI 3 was significantly associated with FP recanalization (aOR = 2.32, *p* < 0.001). Significant controlling covariables were comorbidity of dyslipidemia (aOR = 1.24, *p* < 0.05), treatment with intravenous thrombolysis (aOR = 0.85, *p* < 0.05) and adverse treatment event of intracranial hemorrhage (aOR = 0.62, *p* < 0.05) and clot migration embolization (aOR = 0.4, *p* < 0.001). In the mediator pathway of Model B, groin puncture to recanalization time was significantly associated with FP recanalization (OR = 0.58, *p* < 0.001). Significant controlling covariable was full recanalization mTICI 3 (OR = 0.89, *p* < 0.001). An overview of the logistic and linear regression models is given in Fig. 2**.**

### Mediation analysis

**Fig. 3:**
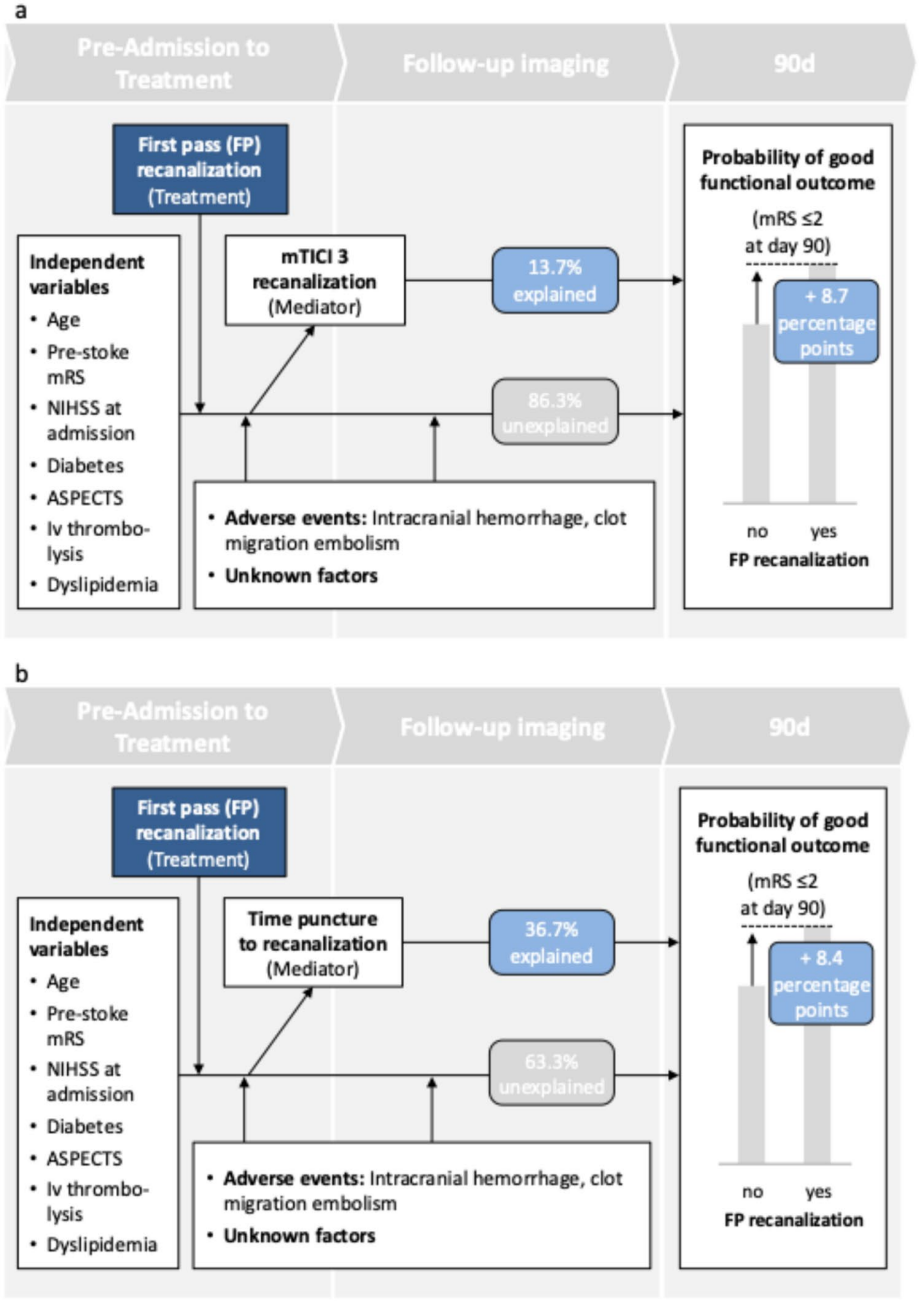
Mediation layout Model A (Mediator mTICI 3 vs. 2b) and Model B (Mediator groin puncture to recanalization time). Schematic model overview of the mediaton analysis, **a** mediator full recanalization, **b** mediator time groin puncture to recanalization. Abbreviations: NIHSS, National Institutes Health Stroke Scale; ASPECTS, Alberta Stroke Program Early CT Score; mRS, modified Rankin Scale; Iv thrombolysis, intravenous thrombolysis; FP first pass; mTICI modified thrombolysis in cerebral infarction

The purpose of the mediation analysis was to investigate the influence of FP recanalization compared to multi-pass recanalization on the probability of good functional outcome mRS 0–2 at day 90 and to quantify the extent to which the FP-related improvement in functional outcome is explained by the degree of recanalization mTICI 2b vs. mTICI 3 (Model A) and by a shorter groin puncture to recanalization time (Model B). The layout of the mediation models is depicted in Fig. 3.

Mediation-based treatment effect estimation in the mediation Model A showed a FP-related increase of good functional outcome of 8.7%-points (95% CI [6.1%-points–11.2%-points]) for the entire cohort (Table 3**, **Fig. 3). 13.7% (CI [6.9%–21.6%]) of the FP-related improvement in functional outcome was explained by the degree of recanalization (mTICI 2b vs. mTICI 3). In mediation Model B with the groin puncture to recanalization time as mediator, mediation-based treatment effect estimation showed a similar FP-related increase of good functional outcome of 8.4%-points (95% CI [4.6%-points–10.9%-points]) whereof 36.7% (CI [19.8%–65.2%]) of the improved functional outcome can be attributed to the time from groin puncture to recanalization.

**Table 3:** Results of the mediation and sensitivity analyses

Mediation analysis A: mediator full recanalization mTICI 3 vs. mTICI 2b				
Outcome: 90 d mRS ≤ 2 Treatment/exposure: first pass recanalization Mediator: mTICI 2b vs. mTICI 3				
Increase of probability of 90 d mRS ≤ 2		Estimate	95% CI	*p*-value
Total effect		8.7%	[6.1%–11.2%]	< 0.001
Direct effect		7.5%	[4.8%–10%]	< 0.001
Effect mediated by full recanalization		1.2%	[0.6%–2%]	< 0.001
% of total effect mediated by FR		13.7%	[6.9%–21.6%]	< 0.001
Sensitivity analysis				
Subgroup	Number of cases	Estimate	95% CI	*p*-value
M1-occlusions	2573			
Total effect		10%	[6.9%–13.4%]	< 0.001
Effect mediated by full recanalization		1.4%	[0.8%–2.1%]	< 0.001
% of total effect mediated by FR		14.4%	[7.5%–18.9%]	< 0.001
M2-occlusions	1134			
Total effect		6%	[1%–11%]	< 0.05*
Effect mediated by full recanalization		Statistically not significant (ACME: *p* > 0.05)		
% of total effect mediated by FR				
Mediation analysis B: mediator groin puncture to recanalization time (min)				
Outcome: 90 d mRS ≤ 2 Treatment/exposure: first pass recanalization Mediator: time groin to recanalization (min)				
Increase of probability of 90 d mRS ≤ 2		Estimate	95% CI	*p*-value
Total effect		8.4%	[4.6%–10.9%]	< 0.001
Direct effect		5.3%	[1.7%–8.4%]	< 0.001
Effect mediated by groin puncture to recanalization time		3.1%	[1.6%–4.9%]	< 0.001
% of total effect mediated by recanalization time		36.7%	[19.8%–65.2%]	< 0.001
Sensitivity analysis				
Subgroup	Number of cases	Estimate	95% CI	*p*-value
M1-occlusions	2573			
Total effect		9.3%	[6.1%–12.8%]	< 0.001
Effect mediated by groin puncture to recanalization time		3.6%	[2%–5.1%]	< 0.001
% of total effect mediated by recanalization time		38.7%	[12.6%–54.6%]	< 0.001
M2-occlusions	1134			
Total effect		5.3%	[1.4%–10.7%]	< 0.05*
Effect mediated by groin puncture to recanalization time		Statistically not significant (ACME: *p* > 0.05)		
% of total effect mediated by recanalization time				

The table shows the results of the mediation analysis A (for full recanalization as mediator), mediation analysis B (for time groin puncture to recanalization as mediator) and the related sensitivity analysis of M1 versus M2 occlusions

*mRS* modified Rankin Scale, *mTICI* modified thrombolysis in cerebral infarction, *FR* full recanalization (mTICI 3), *M1-occlusion* first segment middle cerebral artery occlusion, *M2-occlusion* second segment middle cerebral artery occlusion, *ACME* average causal mediation effect

^*^No mediation effect, because ACME not statistically significant *p* > 0.05

### Sensitivity analysis for subcohorts with M1 and M2 occlusions

Results of the sensitivity analysis of the mediation Model A based on subcohorts with M1 and M2 occlusions indicate that the FP-related improvement in functional outcome is higher for M1 occlusions versus M2 occlusions with a 10%-points (95% CI [6.9%–13.4%]) versus a 6%-points (95% CI [1%–11%]) increase of probability of good functional outcome mRS 0–2. In the M1 cohort, 14.4% (95% CI [7.5%–18.9%]) of the total treatment effect were explained by reperfusion grade mTICI 3 vs. mTICI 2b, for the M2 cohort, the mediation effect was not statistically significant (ACME: *p* > 0.05). Similar applies to the mediation of M1 and M2 occlusion subcohorts for model B, where a shorter recanalization time improves the functional outcome for M1 occlusions by 9.3%–points (95% CI [6.1%–12.8%]) and for M2 occlusions by 5.3%-points (95% CI [1.4%–10.7%]). In the M1 cohort, 38.7% (95% CI [12.6%–54.6%]) of the total treatment effect were explained by a shorter recanalization time, for the M2 cohort, the mediation effect was also not statistically significant (ACME: *p* > 0.05). The results of the mediation analysis are given in Table 3.

## Discussion

Our analysis, based on 3707 patients with acute ischemic stroke who underwent endovascular thrombectomy with successful recanalization (mTICI 2b-3) indicates that the improvement in functional outcomes associated with FP recanalization is not primarily attributable to the higher rates of complete recanalization (mTICI 3) observed in these patients. Instead, the findings suggest that other factors, such as shorter recanalization times and less traumatic interventions, may play a more significant role than achieving complete recanalization. In detail, FP recanalization was associated with a 9%-points increase in probability of good functional outcome mRS 0–2, however, only 14% of this effect was explained by the higher rate of mTICI 3 recanalization compared to 37% explained by a shorter groin puncture to recanalization time observed in the FP cohort.

Previous studies have shown that complete recanalization mTICI 3 is associated with better functional outcome [1, 20] and that patients with FP recanalization have higher rates of mTICI 3 [7], consistent with the baseline characteristics of our cohort. However, our analysis indicates that the impact of mTICI 3 recanalization on functional outcome after FP recanalization is comparatively low and does only explain a small proportion of observed better functional status. This finding suggests two key points: First, mTICI 2b recanalization already achieves a substantial degree of reperfusion, which may provide sufficient cerebral blood flow to support recovery in many patients. Second, a significant advantage of FP recanalization may lie in rapid reperfusion with one pass, which minimizes ischemic damage and procedural trauma.

Consistent with these findings, FP recanalization has been identified as an independent predictor of good functional outcomes [21]. Zaidat et al. [2] proposed several mechanisms to explain the improvement in outcomes associated with FP thrombectomy: (1) Reduced procedural duration minimizes ischemic exposure of brain tissue, thereby limiting infarct progression. (2) Fewer device passes decrease the risk of endothelial injury, vessel dissection, and distal embolization, which are potential precursors to intracerebral hemorrhage. (3) FP recanalization is less likely to disrupt existing collateral blood flow, which is critical for maintaining perfusion in penumbral regions.

Shorter procedure times (groin puncture to recanalization) in FP patients were observed in previous studies [21]. Similarly, our study demonstrated significantly reduced procedure times in the FP cohort compared to the multi-pass cohort (29 min vs. 51 min, respectively). In our mediation analysis, these shorter procedure times accounted for a substantial portion of the improved outcomes observed in the FP cohort.

Higher rates of symptomatic intracranial hemorrhage following multiple retrieval attempts have been described previously [22, 23]. This observation aligns with our findings, where adverse events such as dissections (2% vs. 1%; *p* < 0.05), vasospasms (6% vs. 4%; *p* < 0.05), and intracranial hemorrhages (4% vs. 2%; *p* < 0.001) were more frequent in the multi-pass cohort than in the FP cohort. Notably, the actual differences may be underestimated, as subtle vascular trauma, minor bleeding, and vasospasms are often challenging to detect.

Another potential explanation for the observed improved outcomes is the reduced risk of clot fragmentation associated with fewer recanalization attempts, which may lead to fewer embolic infarcts in distal perfusion territories [24]. Additionally, other yet unidentified factors may contribute to the advantages of FP recanalization, independent of the mTICI score.

In the sensitivity analysis of both Models A and B, a significant mediation effect is observed for M1 occlusions only, while for M2 occlusions, the average mediation effect is not statistically significant (*p* > 0.05). This observation aligns with the results of the multivariable logistic and linear regression analysis, which demonstrated no significant association between the degree of recanalization (mTICI 2b vs mTICI 3; Model A) and groin puncture to recanalization time (Model B) with functional outcomes in M2 occlusions. This is in accordance with previous studies which demonstrate a decreasing benefit of mTICI 3 compared to mTICI 2b for distal occlusions [9, 25]. A possible explanation for the observed lower average treatment effect of mTICI 3 in the M2 cohort is the smaller volume of hypoperfused brain tissue associated with M2 occlusions compared to M1 occlusions. Consequently, the average treatment effect of full recanalization versus partial recanalization and shorter procedure time may also be lower. Additionally, the shorter pathways over the convexity in M2 occlusions may allow collateral circulation to more effectively compensate for partial vessel occlusions. As a result, these patients may still achieve good or even excellent clinical outcomes with mTICI 2b recanalization [26] and longer groin puncture to recanalization times.

In our study, the observed improvement in outcomes associated with FP recanalization compared to multi-pass procedures was less attributable to the higher rates of mTICI 3 recanalization and more strongly linked to a shorter groin puncture to recanalization time. This raises the question of whether mechanical thrombectomy techniques and devices should prioritize achieving successful FP recanalization (mTICI 2b-3) in a short recanalization time over complete recanalization (mTICI 3) [23].

In multivariable logistic regression, full recanalization mTICI 3 and FP recanalization were important positive predictors for good functional outcome. For FP recanalization this relation is even stronger in univariable logistic regression which is also reflected in recent literature [27]. The most important negative predictors for good functional outcome were a long groin puncture to recanalization time and the occurence of intracranial hemorrhage, which may explain part of the benefits of FP thrombectomy over multi-pass recanalization. Among other critical pre-treatment predictors of positive outcomes were younger age, low baseline mRS, and absence of diabetes mellitus, aligning with current evidence [28, 29]. Interestingly, for predicting complete recanalization, the presence of dyslipidemia seems to improve the degree of recanalization in stroke patients with large vessel occlusions. Elevated cholesterol levels, particularly low-density lipoprotein (LDL), may contribute to the formation of more organized, lipid-rich plaques. These types of clots may respond better to mechanical thrombectomy techniques due to their structure, potentially making the clot easier to retrieve compared to more fibrin-rich clots or making an increased response to thrombolytics. The strongest factor in the multivariable regression model predicting full recanalization is the FP effect which is also in harmony with current literature [7].

Our findings suggest that optimizing thrombectomy procedures for rapid FP reperfusion (mTICI ≥ 2b) may lead to better outcomes than pursuing complete reperfusion (mTICI 3) through multiple passes. This has direct implications for procedural strategies and device development. Techniques that facilitate efficient clot removal in a single attempt—such as the use of balloon-guide catheters, larger aspiration catheters [30], or combined approaches like SAVE [31]—may be prioritized. Furthermore, device designs emphasizing effective clot integration and capture in the FP (e.g., stent retrievers with tailored radial force or flexible distal ends) may enhance procedural success. In clinical workflows, minimizing groin puncture to recanalization times through team training and optimized logistics may be as important as the pursuit of complete reperfusion. While mTICI 3 remains a desirable goal, our data suggest that early and safe reperfusion—achieved in the FP—should be a key target for both procedure and device optimization.

Our study had the following limitations: First, for mediation analysis to yield valid results, there must be no unmeasured confounding between the parameters in the proposed causal model. This is a strong assumption, particularly given the complexity of interconnected biological processes, which are not fully understood and may vary between patients. Nonetheless, we believe that the impact of unmeasured confounding has been minimized, as key predictors of functional outcomes after MT were accounted for in the design of the mediation model. Second, the mediation findings from our specific cohort acquired in a retrospective analysis may have other mediating mechanisms than groups with other demographics or risk profile. The fact that our cohort was relatively large and that it was a multicenter study contradicts this. Third, all clinical parameters including mRS and NIHSS were site reported parameters that might suffer from site related bias due to limited inter- and intra-rater reliability and we lack information why specific thrombectomy devices were used. Fourth, only cases with complete data were included in the analysis (n = 5991 out of 13,082 patients with stroke and MT enrolled). Excluding patients with missing data (including those lost to follow-up) may introduce bias into the reported results and potentially limit the generalizability of the findings. Fifth, the decision between pursuing FP recanalization and adopting a multi-pass approach is not one that the interventionalist can make entirely proactively, as it is influenced by factors that are not fully controllable in advance, such as vascular anatomy, clot composition, and procedural circumstances.

In future studies incorporating onset to recanalization time and stratifying between FP mTICI 2b and stepwise progression to mTICI 3 may help to elucidate the additional benefit of further thrombectomy maneuvers.

## Conclusion

Our study emphasizes the critical role of FP compared to multi-pass recanalizations in achieving good functional outcomes after thrombectomy. While only a small proportion of this FP-related improvement in outcome was attributable to higher rates of complete recanalization mTICI 3 observed in FP procedures, shorter procedure times explained a larger proportion of the beneficial effect. Results suggest that the primary advantage of FP recanalization lies in its ability to deliver rapid reperfusion with reduced procedural duration and minimal vascular trauma, which are crucial factors for patient recovery. These insights highlight the importance of minimizing the number of thrombectomy attempts and procedure times and may help to optimize treatment strategies and device design by prioritizing early and efficient reperfusion after the first pass. 

## Abbreviations

ACME: Average causal mediation effect
ADE: Average direct effect
ASPECTS: Alberta Stroke Program Early CT Score
EVT: Endovascular therapy
FP: First pass
FR: Full recanalization
GSR: German Stroke Registry
LVO: Large vessel occlusion
mRS: Modified Rankin Scale
MT: Mechanical thrombectomy
mTICI: Modified thrombolysis in cerebral infarction
NIHSS: National Institutes of Health Stroke Scale
T-FPE: True first pass effect
